# Assessment of the knowledge, attitude, and future practices toward the concept of premarital screening among unmarried Lebanese adults: A cross-sectional study

**DOI:** 10.1371/journal.pgph.0006009

**Published:** 2026-02-23

**Authors:** Omar Al Jassem, Karim Kheir, Rami Rifi, Fatima Fayad, Rayan Kassir, Pascale Salameh

**Affiliations:** 1 Faculty of Medicine, Lebanese University, Beirut, Lebanon; 2 AZM Center for Research in Biotechnology and its Applications, Doctoral School for Sciences and Technology, Lebanese University, Tripoli, Lebanon; 3 Department of Pathology and Laboratory Medicine, American University of Beirut Medical Center, Beirut, Lebanon; 4 Faculty of Pharmacy, Lebanese University, Beirut, Lebanon; 5 Gilbert and Rose-Marie Chagoury School of Medicine, Lebanese American University, Beirut, Lebanon; 6 Department of Primary Care and Population Health, University of Nicosia Medical School, Nicosia, Cyprus; 7 Institut National de Santé Publique d’Épidémiologie Clinique et de Toxicologie-Liban (INSPECT-LB), Beirut, Lebanon; University of Balamand Faculty of Health Sciences, LEBANON

## Abstract

Premarital screening (PMS) is an intervention designed to reduce the burden of genetic and sexually transmitted diseases. Understanding individuals’ knowledge, attitudes and future practices (KAP) toward PMS is essential for promoting informed decision-making in marriage planning. This study aims to assess the KAP of unmarried Lebanese individuals toward PMS and to identify the factors associated with these constructs. A cross-sectional web-based survey was conducted in Lebanon between April and May 2025, using snowball sampling through social media platforms. The survey comprised sociodemographic questions and three sections to assess the KAP toward PMS. Descriptive statistics summarized participants’ characteristics and outcomes, while independent t-tests, ANOVA, and multiple linear regression analyses examined associations and predictors. A total of 422 unmarried participants (mean age: 23.34 ± 3.62 years) completed the survey. The mean knowledge and attitude scores were 17.86 ± 3.25 and 37.89 ± 3.59, respectively. Most participants (97.4%) indicated that they would carry out PMS, and 96.7% disagreed with consanguineous marriage. Higher knowledge scores were found among participants working in the healthcare field (p < 0.001), those who had heard about PMS (p < 0.001), and those who knew its objectives (p < 0.001). Females (p = 0.034), individuals with a history of genetic disease (p < 0.001), and those aware of PMS (p = 0.002) had significantly higher attitude scores. Multiple linear regression analysis identified working in the healthcare field (p = 0.002), marital status (p = 0.006), and awareness of PMS (p = 0.003) as significant predictors of higher knowledge scores. For attitude scores, higher knowledge (p < 0.001) and intention to carry out PMS (p = 0.040) emerged as significant independent predictors. This study revealed a moderate level of knowledge and a good attitude toward PMS among unmarried Lebanese individuals, highlighting the importance of targeted interventions to enhance PMS concepts.

## Introduction

Premarital screening (PMS) is a public health initiative designed to identify and reduce the risk of genetic diseases (GD) and sexually transmitted diseases (STDs), particularly among couples planning to get married. In many countries, particularly those with high rates of consanguineous marriages, PMS is viewed as a preventive tool that enables informed reproductive decisions and can significantly reduce the incidence of inherited disorders such as thalassemia and sickle cell anemia and STDs [1,2]. While many countries in the Middle East and North Africa (MENA) region have introduced PMS programs to mitigate genetic risks, evidence regarding their effectiveness remains mixed [3]. A recent review of the effectiveness of premarital screening programs in the Middle Eastern countries indicated that these programs did not achieve the stated objectives in discouraging at-risk marriages and lowering genetic diseases prevalence except in some countries providing prenatal detection and therapeutic abortion [4].

Despite the availability and importance of PMS services, awareness, knowledge, and acceptance of these programs can vary widely depending on sociocultural, educational, and religious contexts. Studies from Saudi Arabia, Oman, Jordan, and Qatar have consistently shown that while the general attitude toward PMS is positive, knowledge about its objectives, scope, and implications remains inadequate, particularly among young adults [5–8]. For instance, although over 90% of university students in Oman recognized the value of PMS, only about half of them believed it should be mandatory, and misconceptions about genetic inheritance and testing accuracy remain a significant issue [6]. Similar findings were observed in Jordan, where students demonstrated favorable attitudes, but limited understanding of specific disease risks and testing protocols [7]. Recent data from Saudi Arabia indicate that although awareness toward PMS is high (>90% agree on the need for awareness), only around 6–7% of unmarried individuals know which genetic diseases are included in the mandatory screening program [9].

In Lebanon, where consanguineous marriages remain culturally embedded in some communities and where health education on genetic risks is not uniformly accessible, evaluating young adults’ perspectives on PMS is critical. University students, as a key demographic nearing marital age, represent an important population for assessing the tolerance and willingness to undergo public health interventions related to PMS. Yet, data from Lebanon on this subject remains unavailable.

This study aims to fill this gap by evaluating the knowledge, attitude, and future practices toward PMS among unmarried individuals in Lebanon. This research seeks to inform the development of culturally appropriate health education campaigns and strengthen policy efforts to promote informed marital choices and reduce the burden of preventable hereditary diseases and STDs.

## Methods

### Ethical statement, considerations and procedure

This study was conducted following the approval of the Institutional Review Board of the Lebanese Hospital Geitaoui–University Medical Center with approval ID number: 2025-IRB-022. This research was conducted in accordance with the Code of Ethics of the World Medical Association (Declaration of Helsinki III). Prior to participating in this web-based survey, participants were presented with a consent form on the first page. This form provided detailed information about the study’s purpose and aim, scope, data usage, privacy measures taken to ensure data confidentiality and anonymity, and data management procedures. Participants were required to read and agree to the terms outlined in the consent form, indicating their voluntary participation and understanding of the study before proceeding further. Respondents were assured of the confidentiality, privacy, and anonymity of their information. Furthermore, participants were under no obligation and had the freedom to withdraw from the study at any point. Consent was obtained through a “Yes/No” question stating: “Do you agree to participate in this study?” All collected data are confidential and are solely accessible by the team for research purposes.

### Study design

The present study is a cross-sectional descriptive, web-based survey design and was conducted in Lebanon during the period 27 July 2025–10 August 2025. Data was collected through an online questionnaire created using Google Forms, with all items marked as required. Participants were recruited using the snowball sampling technique, primarily through the dissemination of the survey link on various social media platforms such as Facebook and WhatsApp. As a result, only fully completed questionnaires were submitted and included in the final dataset (N = 422). No missing data were observed.

### Participants

Participants in this study were recruited from all eight governorates of Lebanon, ensuring broad geographic representation and enhancing the generalizability of the findings. Individuals were considered eligible for participation if they were unmarried, aged 18 years and above, and residing in Lebanon for more than ten years. Individuals who declined to provide consent or who did not meet the inclusion criteria were excluded from the study. Each participant could only fill the questionnaire once, as respondents were required to sign in with a Google account.

### Sample size

The minimum sample size was calculated using G*Power software version 3.1.9.7 for Windows (Heinrich Heine University Düsseldorf, Germany). As the primary dependent variables are quantitative, multiple regression analyses were planned to assess their correlates. Assuming an effect size of f^2^ = 0.05 (small effect size) and anticipating a squared multiple correlation of R^2^ = 0.05 (deviation from 0) for the omnibus test of multiple regression, the minimum required sample size was determined to be n = 415, based on an alpha level of 5%, a statistical power of 80%, and the inclusion of 20 predictors in the model.

### Study population and collection of samples

The survey instrument was developed through a rigorous three-phase process. In the first phase, items were generated based on an extensive review of the relevant literature, with several questions adapted from previously published instruments [1,6,8]. In the second phase, the questionnaire underwent content and face validation through review by an expert panel consisting of a genetic counselor, an infectious diseases expert, and an epidemiologist from the Lebanese University. This review ensured that the questionnaire comprehensively assessed participants’ knowledge, attitudes, and future practice regarding PMS and GD. In the third phase, pilot testing was carried out with 35 university students to evaluate the questionnaire’s clarity, understandability, and overall acceptability, as well as to modify and update certain linguistic and succinct issues. Feedback from this pilot testing led to minor modifications, including the revision of three questions and the removal of one question. The final questionnaire comprised four sections. The first section included information on participants’ characteristics, such as age, gender, sociodemographic, economic status, and questions about personal and history of hereditary diseases and STDs inside their families. The second section measured participants’ knowledge about PMS through 14 questions with true/false and single-best-answer formats. Correct and incorrect answers were awarded one point and zero points, respectively, with the total knowledge score computed as the sum of correct responses. In the third section, all the questions were related to the attitude of the participants for PMS through 9 items, each rated on a five-point Likert scale, ranging from “strongly disagree” to “strongly agree.” The fourth section evaluated participants’ future practices through four scenario-based questions addressing intentions toward PMS, preferences for consanguineous marriage, decisions regarding marriage in the context of genetic abnormalities, and decisions toward the disclosure of sexually transmitted diseases. For the second and third sections of the questions, knowledge and attitude were less than 60% considered poor, 60–79% considered moderate, and 80% and above considered good according to Bloom’s cut-off [10]. The original survey was developed in English and translated into Arabic using back-to-back translation by bilingual health science experts to ensure accuracy and preserve the intended meaning. In order to assess the reliability and validity of the developed questionnaire, the newly-modified Arabic version was piloted on 35 randomly selected participants.

### Statistical analysis

The collected data were entered into Microsoft Excel and analyzed using the Statistical Package of the Social Sciences (SPSS) v.25. Descriptive statistics, including means, standard deviations, frequencies, and percentages, summarized the sociodemographic and outcome variables. The scores for knowledge and attitude were computed by adding the scores of their respective items. For the knowledge section, correct answers were coded as one and incorrect answers as zero across the 14 items. For the attitude section, the total score was computed by adding the scores of the nine Likert-scale items, ranging from one (strongly disagree) to five (strongly agree). The normality of the knowledge and attitude scores was assessed visually using histograms and normal Q-Q plots, confirming the approximate normal distribution of scores within the sample of 422 participants. The reliability of the scales was evaluated using Cronbach’s alpha and the Kaiser-Mexer-Olkia (KMO) measure of sampling adequacy. For the attitude scale, Cronbach’s alpha was 0.698 (95% CI: 0.64-0.75), and the KMO measure was 0.716 (95% CI: 0.68-0.75) (p < 0.001), indicating acceptable internal consistency and adequate sampling adequacy, respectively. For the knowledge scale, Cronbach’s alpha was 0.672 (95% CI: 0.61-0.73) and the KMO measure was 0.800 (95% CI: 0.77-0.83) (p < 0.001), reflecting satisfactory reliability and sampling adequacy. Bivariate analyses, including independent t-tests, ANOVA, and Chi-square tests, examined the associations between knowledge and attitude scores and sociodemographic characteristics. Factors identified as significant in bivariate analysis were further examined using multiple linear regression to identify independent predictors of knowledge and attitude scores. Stepwise regression was employed to refine these models and to identify the most statistically significant predictors in the dataset while minimizing model complexity. This method is known to systematically add or remove variables based on predefined criteria (mainly p-values), enhancing model interpretability and reducing overfitting by excluding redundant or irrelevant variables. Statistical significance was set at a p-value of less than 0.05.

## Results

### Socio-demographic characteristics and the awareness of the study population

Table 1 shows the sociodemographic characteristics of the 422 participants who completed the survey. The mean age was 23.34 ± 3.62 years, ranging from 18 to 44 years, with 74.6% being females. The vast majority of participants were Lebanese (86.7%) respondents. Regarding the residence, 63.3% of participants lived in urban areas. Additionally, concerning the employment status, 54.3% of participants were employed, of which 28.9% worked in the healthcare field. Nearly all participants were either enrolled in or had graduated from university (97.4%). Moreover, most of the participants were single (88.9%), and 11.1% were engaged. Socioeconomically, 78% belonged to the middle-income group, and when considering family relations, 73.9% of participants’ families were not related, while 6.6% were first cousins, and 13.3% were second cousins.

**Table 1:** Sociodemographic characteristics of the participants (N = 422).

		Frequency (n)	Percentage (%)
Gender	Male	107	25.4
	Female	315	74.6
Age	Mean (SD)	23.34 (3.62)	
	Min – Max	18 – 44	
Age	< 25 years	303	71.8
	≥ 25 years	119	28.2
Nationality	Lebanese	366	86.7
	Syrian	12	2.8
	Palestinian	42	10.0
	Egyptian	2	0.5
Residence place	Rural	267	63.3
	Urban	155	36.7
Employment status	Employee	175	41.5
	Self employed	54	12.8
	Unemployed	193	45.7
Work status	No	193	45.7
	Yes	229	54.3
Work: Healthcare field	No	300	71.1
	Yes	122	28.9
Educational level	Secondary school	11	2.6
	University	411	97.4
If you are currently in university, what is your current year of study?	First year	27	8.8
	Second year	46	15.0
	Third year	70	22.8
	Fourth year	35	11.4
	Fifth year or more	129	42.0
Marital status	Single	375	88.9
	Engaged	47	11.1
How you classify your socioeconomic status?	Low socioeconomic status	47	11.1
	Middle socioeconomic status	329	78.0
	High socioeconomic status	46	10.9
What is the type of relation between your parents?	Not related at all	312	73.9
	First cousins (the couple's parents are brother and sisters)	28	6.6
	Second cousins (the couple's parents are cousins)	56	13.3
	More distant relation	26	6.2

Concerning the awareness and experience of participants regarding PMS and GD, as shown in Table 2, more than half of the participants (59.5%) reported no family history of GD, while 28.0% confirmed a positive family history, and 12.6% were unsure about the presence of a familial GD. In addition, regarding personal health history, only 8.3% reported a positive history. Nearly all participants (99.5%) declared no personal history of STDs, with only 0.5% indicating a history of STDs.

**Table 2:** Awareness and experience related to genetic diseases and premarital screening.

		Frequency (n)	Percentage (%)
Family history of genetic disease?	No	251	59.5
	Yes	118	28.0
	I don’t know	53	12.6
Personal history of genetic disease?	No	387	91.7
	Yes	35	8.3
Personal history of sexual transmitted diseases?	No	420	99.5
	Yes	2	0.5
Have you heard about “Premarital screening” or “genetic testing”?	No	38	9.0
	Yes	384	91.0
Do you know the objectives of “Premarital screening”?	No	66	15.6
	Yes	356	84.4
Have you ever heard about screening for Sexually Transmitted Diseases?	No	4	0.9
	Yes	418	99.1
If yes, what is the main source of your information?	TV	54	12.8
	Family and Friends	158	37.4
	Academic courses	256	60.7
	Social media	197	46.7
	Health care facility	97	23.0
	Newspaper / Journals and articles	12	2.8

The majority of participants (91.0%) confirmed having heard about “premarital screening” or “genetic testing”. Moreover, 84.4% of participants knew the objectives of PMS. The awareness related to the screening for STDs was very high, with 99.1% of participants having heard about it.

When asked about the main sources of information related to PMS and GD, 60.7% cited academic courses as their primary source. Furthermore, social media platforms were reported as a source of information by 46.7% of participants, followed by family and friends (37.4%), health care facilities (23.0%), and television (12.8%). Newspapers, journals, and articles accounted for 2.8% of information sources.

### Assessment of Knowledge and its associated factors regarding PMS and GD

S1 Table shows the responses of participants on knowledge-related questions about PMS and GD. Most participants correctly identified that PMS targets GD (86.5%) and STDs (77.0%), with only 8.1% falsely believing that PMS screens cancer, and 5.0% indicated a lack of knowledge. Regarding GD targeted by PMS, the majority appropriately identified thalassemia (87.0%) and SCD (79.0%) as targets. G6PD deficiency was recognized by 63.5% of respondents, while 52.7% and 55.8% mistakenly included cardiac diseases and diabetes, respectively. Notably, 58.7% of participants indicated they did not know which GD were targeted.

When asked about their awareness toward tests included in PMS, 43.8% answered affirmatively. The generality of participants (98.3%) recognized that consanguinity increases the risk of having a child with GD. Furthermore, 89.1% and 90.3% understood that PMS can reduce the occurrence of GD and STDs, respectively. Concerning the accuracy of genetic testing, 49.1% identified that the latter is not always 100% accurate. Moreover, 55.5% of participants correctly reported that genetic tests do not need to be repeated annually.

Regarding familial GD risk, 92.9% correctly stated that a family history of a GD does not mean that every family member will have the disease. Most participants (93.8%) correctly identified that the appropriate time for genetic testing is before marriage during the engagement period. Additionally, when asked about which sectors of society should be educated about GD, 91.2% selected young people preparing for marriage, 57.6% chose teenagers/high school students, 54.3% chose parents, and 45.3% selected families with known GD.

Moreover, 65.2% of participants correctly answered that GD cannot be cured, whereas 14.7% mistakenly believed they can be, and 20.1% were unsure. Furthermore, 90.0%, 78.0%, and 61.1% of participants appropriately identified sexual intercourse, blood contact, and intravenous drug abuse, respectively, as sources of transmission for HIV, HBV, and HCV, while 17.8% incorrectly identified genetic inheritance as a source. When asked about preventive methods for these infections, 71.6% identified avoiding sharing needles, 59.7% cited the use of condoms, 55.7% mentioned abstinence, and 18.2% noted the use of pills or drugs as preventive measures.

As seen in S2 Table, the mean knowledge score was 17.86 ± 3.25 over 24 with a median of 18.00. These scores ranged from a minimum of 5 to a maximum of 24. The 25^th^ percentile was 16.00, the 50^th^ percentile (median) was 18.00, and the 75^th^ percentile was 20.00.

Table 3 shows the associations between various sociodemographic and experiential factors and knowledge scores among participants. Participants aged 25 years and above had a slightly higher mean knowledge score (18.30) compared to those younger than 25 years (17.69), although the difference was not statistically significant (p = 0.080). Knowledge scores did not significantly differ by gender, nationality, or religion. Similarly, residence location (rural vs. urban) and socioeconomic status did not show significant associations with knowledge scores.

**Table 3:** Factors associated with knowledge scores.

		N	Mean	SD	Effect size	P-value
Age	< 25 years	303	17.69	3.31	d = 0.19	0.080
	≥ 25 years	119	18.30	3.08		
Gender	Male	107	17.66	3.42	d = 0.08	0.470
	Female	315	17.93	3.20		
Nationality	Lebanese	366	17.80	3.31	d = 0.14	0.314
	Other	56	18.27	2.81		
Religion: Christianity	No	390	17.83	3.30	d = 0.11	0.554
	Yes	32	18.19	2.56		
Religion: Islam	No	37	18.35	2.51	d = −0.17	0.337
	Yes	385	17.81	3.31		
Residence place	Rural	267	17.76	3.26	d = 0.08	0.408
	Urban	155	18.03	3.24		
Work status	No	193	17.51	3.47	d = 0.20	**0.044**
	Yes	229	18.15	3.03		
Work: Healthcare field	No	300	17.48	3.34	d = 0.41	**<0.001**
	Yes	122	18.79	2.83		
Educational level	Secondary school	11	16.36	2.42	d = 0.47	0.122
	University	411	17.90	3.26		
Marital status	Single	375	18.02	3.23	d = −0.43	**0.005**
	Engaged	47	16.62	3.21		
How you classify your socioeconomic status?	Low socioeconomic status	47	17.68	2.85	η² = 0.008	0.192
	Middle socioeconomic status	329	17.99	3.26		
	High socioeconomic status	46	17.09	3.54		
Family history of genetic disease?	No	251	17.92	3.23	η² = 0.001	0.806
	Yes	118	17.84	3.30		
	I don’t know	53	17.60	3.31		
Personal history of genetic disease?	No	387	17.83	3.24	d = 0.12	0.485
	Yes	35	18.23	3.42		
Personal history of sexual transmitted diseases?	No	420	17.85	3.26	d = 0.66	0.352
	Yes	2	20.00	1.41		
Have you heard about “Premarital screening” or “genetic testing”?	No	38	15.24	3.89	d = 0.91	**<0.001**
	Yes	384	18.12	3.07		
Do you know the objectives of “Premarital screening”?	No	66	15.71	3.72	d = 0.82	**<0.001**
	Yes	356	18.26	3.00		
Have you ever heard about screening for Sexually Transmitted Diseases?	No	4	13.00	6.16	d = 1.52	**0.003**
	Yes	418	17.91	3.19		
If you ever decided to get married, will you carry premarital screening?	No	11	16.45	4.08	d = 0.45	0.147
	Yes	411	17.90	3.22		
Do you prefer consanguineous marriages (marriage between cousins or relatives)?	No	408	17.93	3.23	d = −0.62	**0.024**
	Yes	14	15.93	3.52		

Tests are done using the independent t-test and ANOVA test. Bold: Significance set at 5%. SD: Standard deviation. Min: Minimum value. Max: Maximum value.

Regarding employment, those who were currently employed had significantly higher knowledge scores (mean = 18.15) than those who were not (mean = 17.51; p = 0.044). Additionally, participants working in the healthcare field had significantly higher knowledge scores (mean = 18.79) compared to those working in other fields (mean = 17.48; p < 0.001).

Interestingly, the education level did not show a significant association with knowledge scores, although participants at the university level tended to have slightly higher scores. Marital status was significantly associated with higher knowledge scores, with single respondents scoring higher on average (mean = 18.02) compared to engaged participants (mean = 16.62; p = 0.005).

Furthermore, while personal and family history of GD did not show a significant association with knowledge scores, these scores were significantly higher among participants who had heard about PMS (mean = 18.12 vs. 15.24; p < 0.001), those who knew its objectives (mean = 18.26 vs. 15.71; p < 0.001), and those who had heard about STDs screening (mean = 17.91 vs. 13.00; p = 0.003). Moreover, participants who expressed disagreement with consanguineous marriage had significantly higher knowledge scores (mean = 17.93) compared to those who preferred consanguineous marriage (mean = 15.93; p = 0.024).

The results of the multiple linear regression analysis examining factors independently associated with knowledge scores related to PMS and GD are shown in Table 4. Both an enter model and a stepwise model were conducted to identify significant predictors. In the enter model, age and employment in the healthcare field were positively associated with knowledge scores (p = 0.041 and p = 0.007, respectively), suggesting that older participants, as well as those involved in the healthcare sector, had slightly higher knowledge scores. Furthermore, marital status was also significantly associated with knowledge scores, with single individuals scoring higher than their engaged counterparts (p = 0.007). Awareness and understanding of PMS were also significant predictors. Participants who had heard about PMS had higher knowledge scores (p = 0.002), as did those who knew the objectives of PMS (p = 0.001). Preference for consanguineous marriage did not reach significance (p = 0.073), although a trend toward lower knowledge scores was detected among those who preferred consanguineous marriage.

**Table 4:** Multiple linear regression for the factors affecting the knowledge score.

Variables	Enter Model						Stepwise Model					
	Unstandardized Coefficients	P.value	95.0% Confidence Interval for B		Effect size (ENTER): partial r	Effect size (ENTER): f²	Unstandardized Coefficients	P.value	95.0% Confidence Interval for B		Effect size (STEPWISE): partial r	Effect size (STEPWISE): f²
	B		Lower Bound	Upper Bound			B		Lower Bound	Upper Bound		
(Constant)	13.711	<0.001	9.558	17.864			16.050	<0.001	14.602	17.498		
Age	0.726	0.041	0.030	1.422	0.100	0.010					—	—
Work status	-0.427	0.274	-1.194	0.340	-0.054	0.003					—	—
Work: Healthcare field	1.103	0.007	0.305	1.900	0.132	0.018	1.017	0.002	0.364	1.670	0.148	0.022
Marital status	-1.275	0.007	-2.206	-0.344	-0.131	0.017	-1.297	0.006	-2.225	-0.369	-0.133	0.018
Have you heard about “Premarital screening” or “genetic testing”?	1.849	0.002	0.672	3.027	0.150	0.023	1.820	0.003	0.641	2.998	0.147	0.022
Do you know the objectives of “Premarital screening”?	1.562	0.001	0.611	2.513	0.157	0.025	1.543	0.001	0.600	2.486	0.155	0.025
Do you prefer consanguineous marriages (marriage between cousins or relatives)?	-1.490	0.073	-3.120	0.139	-0.088	0.008					—	—
Educational level	1.213	0.200	-0.644	3.070	0.063	0.004					—	—
How do you classify your socioeconomic status?	-0.399	0.209	-1.021	0.224	-0.062	0.004					—	—
Dependent: Knowledge score												

In the stepwise model, significant predictors of higher knowledge scores included working in the healthcare field (p = 0.002), marital status (p = 0.006), awareness toward PMS (p = 0.003), and knowing the objectives of PMS (p = 0.001). These findings demonstrate that knowledge about PMS is significantly influenced by information exposure, marital status, and professional experience in healthcare, highlighting key areas for targeted educational interventions.

### Assessment of attitudes and its associated factors towards PMS and GD

S3 Table demonstrates that the majority (98.3%) of participants agreed that awareness about PMS before marriage is important. Moreover, 88.9% of participants also agreed that GD can have a psychological burden on families, and 84.1% recognized the potential economic burden of these conditions.

A considerable proportion (76.1%) agreed that genetic testing could lead to the denial of marriage among some couples. In addition, the generality of participants (89.3%) rejected the notion that genetic testing will do more harm than good for society, with only 10.7% agreeing with this statement. Regarding the role of laws, 83.4% of participants agreed that laws should obligate future couples to undergo PMS and genetic counseling. In terms of religious and cultural beliefs, 69.4% of respondents agreed that PMS does not interfere with a belief in destiny.

The necessity of screening for STDs before marriage was strongly affirmed by 95.3% of participants. Finally, 64.0% of respondents agreed that decisions regarding marriage in the context of STDs detection should be left to the freedom of the couple.

S4 Table shows the distribution of attitude scores related to PMS and genetic testing among the 422 participants. The mean attitude score was 37.89 ± 3.59 over 45 with a median of 38.00. Scores ranged from a minimum of 27 to a maximum of 45. The 25^th^ percentile was 36.00, the 50^th^ percentile (median) was 38.00, and the 75^th^ percentile was 41.00.

Table 5 shows that the gender was significantly associated with attitudes, as females had higher attitude scores (mean = 38.10) compared to males (mean = 37.25; p = 0.034). Although attitude scores did not vary significantly by nationality (p = 0.080) or residence (p = 0.090), participants working in the healthcare field had marginally higher attitude scores (mean = 38.35) than those in other fields (mean = 37.70), though this did not reach significance (p = 0.090).

**Table 5:** Factors Associated with Attitude.

		N	Mean	SD	Effect size	P.value
Age	< 25 years	303	37.77	3.56	d = 0.12	0.262
	≥ 25 years	119	38.20	3.64		
Gender	Male	107	37.25	3.99	d = 0.24	**0.034**
	Female	315	38.10	3.42		
Nationality	Lebanese	366	38.01	3.46	d = -0.25	0.080
	Other	56	37.11	4.30		
Religion: Christianity	No	390	37.89	3.60	d = -0.00	0.982
	Yes	32	37.88	3.54		
Religion: Islam	No	37	38.22	3.50	d = -0.10	0.561
	Yes	385	37.86	3.60		
Residence place	Rural	267	37.66	3.63	d = 0.17	0.090
	Urban	155	38.28	3.49		
Work status	No	193	37.62	3.64	d = 0.14	0.161
	Yes	229	38.11	3.54		
Work: Healthcare field	No	300	37.70	3.57	d = 0.18	0.090
	Yes	122	38.35	3.60		
Educational level	Secondary school	11	37.91	3.67	d = -0.01	0.985
	University	411	37.89	3.59		
Marital status	Single	375	37.90	3.59	d = -0.04	0.804
	Engaged	47	37.77	3.57		
How you classify your socioeconomic status?	Low socioeconomic status	47	38.83	2.78	η² = 0.009	0.159
	Middle socioeconomic status	329	37.76	3.67		
	High socioeconomic status	46	37.87	3.64		
Family history of genetic disease?	No	251	37.97	3.42	η² = 0.002	0.654
	Yes	118	37.90	3.89		
	I don’t know	53	37.47	3.69		
Personal history of genetic disease?	No	387	37.69	3.60	d = 0.67	**<0.001**
	Yes	35	40.06	2.62		
Personal history of sexual transmitted diseases?	No	420	37.89	3.59	d = 0.17	0.809
	Yes	2	38.50	3.54		
Have you heard about “Premarital screening” or “genetic testing”?	No	38	36.16	3.74	d = 0.54	**0.002**
	Yes	384	38.06	3.53		
Do you know the objectives of “Premarital screening”?	No	66	36.86	3.69	d = 0.34	**0.011**
	Yes	356	38.08	3.54		
Have you ever heard about screening for Sexually Transmitted Diseases?	No	4	36.75	3.40	d = 0.32	0.524
	Yes	418	37.90	3.59		
If you ever decided to get married, will you carry premarital screening?	No	11	35.36	4.92	d = 0.73	**0.018**
	Yes	411	37.96	3.53		
Do you prefer consanguineous marriages (marriage between cousins or relatives)?	No	408	37.94	3.58		0.089
	Yes	14	36.29	3.63		

Tests are done using the independent t-test and ANOVA test. Bold: Significance set at 5%. SD: Standard deviation. Min: Minimum value. Max: Maximum value

Of particular interest, personal history of GD was significantly associated with attitude scores (p < 0.001), as those with a personal history of GD had substantially higher mean attitude scores (40.06) compared to those with a negative history (37.69). Awareness-related variables also showed significant differences: participants who had heard about PMS (mean = 38.06), as well as those who knew its objectives (mean = 38.08), had higher attitude scores than their counterparts (p = 0.002 and p = 0.011, respectively).

In addition, participants intending to carry out PMS had significantly higher attitude scores (mean = 37.96) than their counterparts (mean = 35.36; p = 0.018). However, in this analysis, age, marital status, socioeconomic status, family history of GD, and preference for consanguineous marriage did not show significant associations with attitude scores.

Table 6 presents the results of the multiple linear regression analysis assessing the independent factors associated with attitude scores among participants. Both the enter model and stepwise model were used to identify significant predictors. In the enter model, knowledge score was a significant positive predictor of attitude score (p < 0.001), indicating that higher knowledge was associated with a more favorable attitude. Gender was also significantly associated with attitude scores (p = 0.047), with females having higher attitude scores than males. Nevertheless, while other variables, such as working in the healthcare field and awareness of PMS, showed positive association with attitude scores, they did not reach statistical significance in the enter model.

**Table 6:** Multiple linear regression for the factors affecting the attitude scores.

Variables	Enter Model						Stepwise Model					
	Unstandardized Coefficients	P.value	95.0% Confidence Interval for B				Unstandardized Coefficients	P.value	95.0% Confidence Interval for B			
	B		Lower Bound	Upper Bound	R	Cohen’s f²	B		Lower Bound	Upper Bound	R	Cohen’s f²
(Constant)	28.207	<0.001	24.552	31.861			29.323	<0.001	26.258	32.388	0.271	0.079
Knowledge	0.231	<0.001	0.123	0.339	0.231	0.056	0.271	<0.001	0.170	0.373	0.179	0.033
Gender	0.787	0.047	0.009	1.564	0.098	0.010						
Residence place	0.426	0.229	-0.269	1.120	0.061	0.004						
Work status	0.213	0.613	-0.613	1.039	0.027	0.001						
Work: Healthcare field	0.264	0.571	-0.651	1.178	0.032	0.001						
How you classify your socioeconomic status?	-0.498	0.168	-1.206	0.210	0.072	0.005						
Personal history of sexual transmitted diseases?	0.349	0.887	-4.461	5.159	0.008	0.000						
Have you heard about “Premarital screening” or “genetic testing”?	0.888	0.198	-0.466	2.242	0.064	0.004						
Do you know the objectives of “Premarital screening”?	0.050	0.929	-1.046	1.146	0.004	0.000						
If you ever decided to get married, will you carry premarital screening?	2.066	0.052	-0.015	4.147	0.101	0.010	2.179	0.040	0.103	4.255		

Dependent: Attitude score

The stepwise model identified three significant independent predictors of attitude scores. Knowledge scores, as well as the intent to carry out PMS, were positively associated with attitude scores (p < 0.001 and p = 0.049, respectively).

### Future practices toward PMS and related issues

97.4% of participants indicated that they would carry out PMS if they ever decided to get married. Similarly, 96.7% of respondents expressed a preference against consanguineous marriage. In case of a PMS revealing a chance of having a child affected by a GD, 42.4% of participants indicated that their decision would depend on the probability of the disease, while 17.3% would cancel or discontinue the engagement. Approximately 25.8% stated they would proceed with marriage and opt for in-vitro fertilization (IVF) to selectively avoid having children with inherited disorders. Smaller proportions stated they would continue the engagement and marriage for emotional reasons (7.1%) or without having children (7.3%). Regarding the discovery of an STD during premarital counseling, 69.9% of participants said they would inform their partner, and 68.5% would consult a physician. Conversely, 31.0% would choose to break the relationship, and only 0.2% would hide this information from their partner (S5 Table).

### Influence of knowledge and attitude scores on decision-making in case of GD risk

Overall, while knowledge scores did not vary significantly across most decision scenarios, attitude scores exhibited some notable associations as shown in the Table 7. For the scenario “Decision will depend on the probability of transmitting the disease”, attitude scores were significantly higher among those who chose “No” (mean = 38.21) compared to those who chose “Yes” (mean = 37.46; p = 0.034), while knowledge scores did not differ significantly (p = 0.173).

**Table 7:** Influence of knowledge and attitude scores on decision-making in case of GD risk.

		Knowledge					Attitude			
		N	Mean	SD	P.value	Effect size	Mean	SD	P.value	Effect size
Decision in case of genetic disease: Decision will depend on the probability of transmitting the disease	No	243	18.05	3.20	0.173	d = -0.14	38.21	3.68	**0.034**	d = -0.21
	Yes	179	17.61	3.31			37.46	3.42		
Decision in case of genetic disease: Cancel/ discontinue engagement	No	349	17.81	3.29	0.472	d = 0.09	37.83	3.63	0.428	d = 0.10
	Yes	73	18.11	3.08			38.19	3.39		
Decision in case of genetic disease: Get married and I would go for IVF to selectively have children without the inherited disorder.	No	313	17.65	3.29	**0.023**	d = 0.25	37.62	3.59	**0.009**	d = 0.29
	Yes	109	18.47	3.09			38.66	3.48		
Decision in case of genetic disease: Continue engagement and get married for emotional reasons	No	392	17.97	3.20	**0.011**	d = -0.49	37.99	3.51	**0.032**	d = -0.41
	Yes	30	16.40	3.64			36.53	4.29		
Decision in case of genetic disease: Continue engagement and get marriage without getting children	No	391	17.85	3.27	0.804	d = 0.05	37.86	3.55	0.552	d = 0.11
	Yes	31	18.00	3.08			38.26	4.09		

Tests are done using the independent t-test. Bold: Significance set at 5%. SD: Standard deviation

Meanwhile, in the scenario of choosing to “Cancel/Discontinue engagement”, neither knowledge scores (p = 0.472) nor attitude scores (p = 0.428) demonstrated significant differences between groups. For those who opted to “Get married and go for IVF”, knowledge scores were significantly higher (mean = 18.47) compared to those who did not choose this option (mean = 17.65; p = 0.023). Attitude scores were also significantly higher among the former group (mean = 38.66 vs. 37.62; p = 0.009). Additionally, participants who chose to “Continue engagement and get married for emotional reasons” had significantly lower knowledge scores (mean = 16.40 vs. 17.97; p = 0.011) and lower attitude scores (mean = 36.53 vs. 37.99; p = 0.032) compared to those who did not choose this option.

Finally, no significant differences in knowledge (p = 0.804) or attitude scores (p = 0.552) were observed in the scenario of “Continue engagement and get married without getting children”.

### Correlation between knowledge and attitude

**Fig 1:**
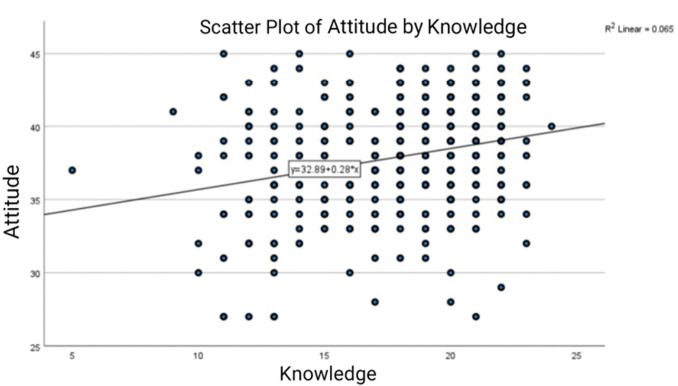
Correlation between Knowledge and Attitude towards PMS.

A scatter plot illustrating the correlation between knowledge scores and attitude scores among participants is shown in Fig 1, with a linear regression line included. The Pearson correlation coefficient was found to be 0.254 (p < 0.001), indicating a statistically significant positive correlation between knowledge and attitude scores. This suggests that participants with higher knowledge levels tend to have slightly better attitude toward PMS and genetic testing. However, the coefficient of determination (R² = 0.065) suggests that knowledge explains only a small proportion of the variance in attitude scores, indicating that other factors may also influence attitude beyond knowledge alone.

## Discussion

While the consanguinity rate is infrequently reported in many countries worldwide, it remains significantly high in the MENA region, where social beliefs, as well as cultural and religious practices, encourage marriage between family members. Among these factors, strengthening family ties, maintaining one’s religious beliefs by choosing a partner of the same religion, as well as prioritizing the family’s wishes through arranged marriage, remain among the most common reasons behind the perpetuation of consanguinity.

To our knowledge, the present research is the first study conducted in Lebanon to assess the knowledge, attitude, and practice among unmarried Lebanese individuals regarding PMS.

### Knowledge toward PMS

Our findings suggest a competent knowledge of participants toward PMS, as most of them (89.1%) stated that PMS aims to reduce the occurrence of GD. This finding is similar to that seen in a study conducted in Oman (88.8%) [6], compared to 85.2%, 80.3%, 79.89%, 69.7%, 65.8%, and 47.3% of participants in studies conducted in Sudan [11], Jordan [12], Yemen [13], Saudi Arabia [14], Iraq [1], and UAE [15] respectively. These results indicate a better level of knowledge among the Lebanese population, compared to other Arab countries.

Nevertheless, despite the fact that 86.5% of participants correctly identified that PMS targets GD, more than half of respondents (58.7%) did not know which GD are actually targeted, and 56.2% reported not knowing the exact tests included in PMS. These findings highlight the need for additional awareness regarding the objectives of PMS, and the GD it aims to screen.

Moreover, our study shows a mean score of 17.86 ± 3.25 over 24 (74.4%) regarding participants’ knowledge of PMS. This score falls in the interval of 60–79%, and reflects, according to Bloom’s cut-off, a moderate level of knowledge among unmarried Lebanese individuals [10].

This highlights the need for additional awareness, through educational campaigns and cultural interventions, to enhance the knowledge toward PMS among the Lebanese youth. Integration of the concepts of PMS among both school and university students is of particular interest, as this step ensures early assimilation of the importance of conduction of premarital screening tests, and the benefits they provide for both the couple and its offspring. Moreover, governmental funds aiming to provide premarital screening tests at reduced fees can help improve awareness toward PMS, as many couples might not consider undergoing PMS simply for financial reasons.

Although our study shows a moderate level of knowledge, this score remains higher among the Lebanese population compared to other Arab countries. For instance, in Yemen, more than half of the participants (58.82%) a had weak knowledge [16], as well as in Egypt [17] and Saudi Arabia [18], where only 30.6% and 9.2% of respondents had satisfactory knowledge toward PMS, respectively. Additionally, our participants demonstrated a better level of knowledge compared to a study conducted in Ghana, where only 15.4% of respondents had adequate knowledge [19].

### Factors associated with knowledge scores

While older participants, as well as those with a university educational level, tended to have higher knowledge scores, no significant association was found between age or educational level and knowledge toward PMS. These findings do not go in line with studies conducted in Saudi Arabia [18], Sudan [11], and Nigeria [20], where participants’ knowledge toward PMS was associated with age and level of education. Consequently, additional studies in Lebanon are needed to elucidate whether age and educational level of participants are actually associated with their level of knowledge toward PMS and genetic counselling.

Additionally, while our study found no association between gender and knowledge score, similarly to the findings of a Qatari study [21], this score was found to be higher among females in studies conducted in Syria [22], Yemen [13], and Saudi Arabia [18]. These findings can be attributed to targeted teaching of females about topics related to marriage in countries where society emphasizes the traditional role of females in being accountable for marriage planning and preparation.

On the other hand, our study shows a significant association between marital status and knowledge scores (p = 0.005), similar to studies conducted in Iraq [1] and Saudi Arabia [18]. Additionally, work status and being in the medical field were also linked to higher knowledge scores, with p-values of 0.044 and <0.001, respectively, which is compatible with the findings of studies done in Qatar [8], and Saudi Arabia [23]. This can be explained by the fact that workers in the healthcare field are more likely to have better awareness about PMS and genetic counseling, compared to those who work in other, non-health-related fields.

Furthermore, higher knowledge scores were remarkably noted among respondents who had previously heard about PMS, similarly to the findings of an Iraqi study [1], as well as among participants who knew the objectives of PMS, those who had heard about screening for STDs, and those who do not prefer consanguineous marriage. Likewise, a Qatari study done in 2022 demonstrated that consanguinity was significantly linked to lower knowledge scores [8], while a study conducted in Egypt showed no significant correlation [17].

### Attitude toward PMS

Among our participants, the attitude toward PMS was strongly positive, with 98.3% agreeing on its importance before marriage. As a matter of fact, to the best of our knowledge, this percentage was found to be the highest among all studies conducted in the Arab world, exceeding the percentage of participants agreeing on the importance of PMS in Saudi Arabia (95.2%) [14], Oman (84.5%) [6], Iraq (83.1%) [1], UAE (79.5%) [15], Libya (79%) [24], and Egypt (76.1%) [17].

In addition, the majority of our participants (89.3%) stated that genetic testing will do more good than harm for society. This highlights a better attitude among Lebanese individuals compared to studies conducted in Qatar [8] and the UAE [15], where only 78% and 59% agreed with this statement, respectively.

Furthermore, 83.4% of participants supported the view of implementing PMS and genetic counseling as a mandatory tool for screening among future couples before marriage, reflecting a positive attitude among participants. While 88.3% of respondents in a Jordanian study agreed with this statement [12], showing even higher positive attitude than participants in our study, only 78.6%, 72.1%, 71.2%, 61.7%, 49.5%, and 47.62% of individuals agreed on making PMS compulsory, according to studies conducted in Iraq [1], Saudi Arabia [14], Sudan [11], Libya [24], Oman [6], and Yemen [13], respectively.

In light of our findings, our participants had a mean attitude score of 37.89 ± 3.59 over 45 (84.2%). This score is greater than 80%, and hence reflects a good attitude toward PMS and genetic counseling according to Bloom’s cut-off [10].

### Factors associated with attitude scores

Our study demonstrated that females had higher attitude scores than males, with a significant p-value of 0.034, which is concordant with the results of a study conducted in Saudi Arabia [25]. Nevertheless, no significant association was found in a study done in Iraq [1]. Hence, future studies are required to confirm whether gender is actually linked to higher attitude scores.

Moreover, greater attitude scores were seen among individuals with a history of GD, most likely due to increased recognition of the negative impact of these conditions (p < 0.001), as well as among those who have previously heard about PMS, those who knew its objectives, and those who were willing to undergo PMS in case of marriage (p = 0.002, p = 0.011, and p = 0.18, respectively). In fact, participants who fulfill these criteria are expected to show better awareness of the positive impact and advantages of PMS and genetic counselling, which translates to higher attitude scores among these individuals.

### Future practice toward PMS

The vast majority of our participants (97.4%) were willing to proceed with PMS before marriage, which shows evidence of a good attitude toward PMS. Likewise, two studies conducted in Saudi Arabia revealed identical findings, where 96.5% [14] and 96.12% [26], of respondents were willing to undergo PMS. Nevertheless, other studies conducted in Sudan [11], Libya [24], Oman [6], and Yemen [13], showed less favorable attitudes toward PMS compared to our study, as only 82.3%, 79%, 69.5%, and 68% of participants expressed their willingness to undergo PMS, respectively.

Additionally, only 3.3% of our participants showed a preference toward consanguineous marriage, compared to 10.5%, 13.5%, and even 50% in Oman [6], Libya [24], and the UAE [15], respectively. These findings highlight a better attitude among the Lebanese population against consanguineous marriage, a practice related to an increased risk of GD, compared to other Arab countries.

In the case of positive PMS findings, only 17.3% of our respondents confirmed that they would end their engagement, while 37.4% and 49.8% of participants stated that they would adopt this approach in Iraq [1] and Jordan [12], respectively. These findings can be explained by the fact that our participants leaned more toward other options, such as deciding to continue the engagement or not based on the probability of disease transmission to the offspring (42.4%), or the ability to perform IVF to selectively have children unaffected by the disorder (25.8%).

### Influence of knowledge and attitude scores on decision-making in case of GD risk

Our study demonstrates that knowledge and attitude scores can affect participants’ decisions toward continuation of engagement and marriage in case of GD risk.

In fact, in the case of GD risk, participants who chose to “get married and go for IVF to selectively have children without the inherited disorder”, as well as those who did not accept to “continue engagement and get married for emotional reasons”, had higher knowledge scores than their counterparts, with significant p-values of 0.023 and 0.011, respectively.

Similarly, in case of GD risk, respondents who were willing to stop marriage irrespective of the probability of disease transmission, as well as those who chose to “get married and go for IVF”, and those who refused to “continue engagement and get married for emotional reasons”, had higher attitude scores, with significant p-values of 0.034, 0.009, and 0.032, respectively.

These findings highlight that higher knowledge and attitude scores are correlated with better decision-making skills among participants before marriage, particularly in the case of GD risk.

### Limitations

The present study has several limitations. For instance, our study relied on the snowballing technique for data collection, which might have led to a non-random selection, due to over-representation of individuals with similar backgrounds and opinions. A more random data collection technique might have provided more accurate results, but could not be performed in our study due to limited resources. Moreover, other unmeasured confounders, such as uneven access to genetic counseling services, as well as uneven health literacy, might have influenced our findings. In addition, self-reporting of participants is considered less precise than face-to-face interviewing, as it increases the bias related to poor understanding and misinterpretation of the questionnaire. Finally, non-response of some individuals has limited our sample size; hence, future studies with a greater sample size are needed in order to reach more representative and generalizable results.

## Conclusion

In summary, the present research is the first to be conducted in Lebanon in order to assess the knowledge, attitude, and practice of Lebanese unmarried individuals regarding PMS. While the mean knowledge score of participants reflected a moderate knowledge toward PMS, this knowledge was higher among the Lebanese population compared to other Arab countries.

In addition, this study shows a good attitude toward PMS, with the majority of participants (83.4%) supporting the implementation of PMS and genetic counseling prior to marriage. Furthermore, the present research shows a promising practice concerning the future of unmarried Lebanese individuals, as 97.4% of participants confirmed their willingness to perform PMS before getting married. According to the medical literature, this percentage is the highest among all other studies conducted in the Arab world. Finally, it was demonstrated through this study that higher knowledge and attitude scores were linked to better decision-making skills before marriage, particularly in the case of presence of a genetic disease risk.

## Supporting information

S1 Table Knowledge of participants regarding premarital screening and genetic diseases. (XLSX)

S2 Table Distribution of Knowledge scores among participants. (XLSX)

S3 Table Attitude of participants toward premarital screening and genetic testing. (XLSX)

S4 Table Distribution of attitude scores among participants. (XLSX)

S5 Table Future Practices of Participants Toward Premarital Screening and Related Issues. (XLSX)

S1 Data Participant data. (XLSX)
